# Differential Expression of IRS-1 and IRS-2 in Uterine Leiomyosarcomas with Distinct Oncogenic Phenotypes: Lack of Correlation with Downstream Signaling Events

**DOI:** 10.1080/1357714021000065387

**Published:** 2002-09

**Authors:** Alfonso Colombatti, Pietro Russo, Marta Cervi, Laura Bogetto, Bruna Wassermann, Fabrizio Mainiero, Paola Spessotto

**Affiliations:** 1 Divisione di Oncologia Sperimentale 2 Centro di Riferimento Oncologico-IRCCS Aviano 33081 Italy; 2 Dipartimento di Scienze e Tecnologie Biomediche and MATI Excellence Center Università di Udine Udine 35100 Italy; 3 Dipartimento di Medicina Sperimentale e Patologia Università La Sapienza Roma Italy

## Abstract

*Purpose:* Insulin receptor substrates (IRSs) are essential for insulin-induced mitogenic effects on several cell types but they also are involved in cell transformation.We investigated whether the differential constitutive expression and potential distinct downstream signaling events of IRS-1 and IRS-2 might be related to discrete tumourigenic phenotypes of three human uterine leiomyosarcoma cell lines, one of which was specifically isolated for the present study.

*Methods and results:* SK-UT-1B egressed effectively from a gellyfied Matrigel matrix and grew as did DMR cells in an anchorage-independent manner in agar and induced rapidly growing tumours in nude mice. On the contrary, SK-LMS-1 cells did not emigrate from Matrigel, neither grew in agar nor were they tumourigenic. IRS-2 was highly expressed in the more malignant cell lines, whereas IRS-1 was present only in SK-LMS-1 cells. However, upon insulin stimulation both IRS- 1 and IRS-2 were tyrosine phosphorylated with a similar kinetic in the respective cell lines; furthermore, after 1 min of insulin stimulation PI3-kinase associated with IRSs and after 2 min Shc was phosphorylated and associated with Grb2 with minor differences detectable among the various cell lines in the duration of phosphorylation and/or in their association irrespective of whether IRS-1 or IRS-2 were expressed.

*Discussion:* Our findings tend to exclude that the malignancy displayed by uterine leiomyosarcomas might be directly linked to the activation of distinct IRS-1- or IRS-2-dependent pathways.


*Sarcoma* (2002) **6**, 89–96

Taylor & Francis healthsciences

**ORIGINAL ARTICLE**

### Differential expression of IRS-1 and IRS-2 in uterine leiomyosarcomas with distinct oncogenic phenotypes: lack of correlation with downstream signaling events

ALFONSO COLOMBATTI[1,2,*], PIETRO RUSSO[1], MARTA CERVI[1], LAURA BOGETTO[1], BRUNA WASSERMANN[1], FABRIZIO MAINIERO[3] & PAOLA SPESSOTTO[1]

[1]*Divisione di Oncologia Sperimentale 2, CRO-IRCCS, 33081 Aviano, Italy,* [2]*Dipartimento di Scienze e Tecnologie Biomediche and MATI Excellence Center, Università di Udine, 35100 Udine, Italy and* [3]*Dipartimento di Medicina Sperimentale e Patologia, Università La Sapienza, Roma, Italy*

**Abstract**

*Purpose:* Insulin receptor substrates (IRSs) are essential for insulin-induced mitogenic effects on several cell types but they also are involved in cell transformation. We investigated whether the differential constitutive expression and potential distinct downstream signaling events of IRS-1 and IRS-2 might be related to discrete tumourigenic phenotypes of three human uterine leiomyosarcoma cell lines, one of which was specifically isolated for the present study.

*Methods and results:* SK-UT-1B egressed effectively from a gellyfied Matrigel matrix and grew as did DMR cells in an anchorage-independent manner in agar and induced rapidly growing tumours in nude mice. On the contrary, SK-LMS-1 cells did not emigrate from Matrigel, neither grew in agar nor were they tumourigenic. IRS-2 was highly expressed in the more malignant cell lines, whereas IRS-1 was present only in SK-LMS-1 cells. However, upon insulin stimulation both IRS-1 and IRS-2 were tyrosine phosphorylated with a similar kinetic in the respective cell lines; furthermore, after 1 min of insulin stimulation PI3-kinase associated with IRSs and after 2 min Shc was phosphorylated and associated with Grb2 with minor differences detectable among the various cell lines in the duration of phosphorylation and/or in their association irrespective of whether IRS-1 or IRS-2 were expressed.

*Discussion:* Our findings tend to exclude that the malignancy displayed by uterine leiomyosarcomas might be directly linked to the activation of distinct IRS-1- or IRS-2-dependent pathways.

### Introduction

Insulin exerts a large series of integrated actions via a receptor-mediated intracellular signaling system.[1] The insulin receptor, a member of the tyrosine kinase growth factor receptor family, is phosphorylated upon insulin binding; then, it induces the phosphorylation of multiple tyrosine residues within the C-terminus of a family of signaling molecules referred to as insulin receptor substrate (IRS) proteins,[2–4] the best characterized of which are IRS-1 and IRS-2. The anchoring of IRS-1 and IRS-2 to the detergent-resistant insoluble fraction corresponding to the cytoskeleton[5–8] facilitates interaction with downstream signaling proteins. The advantage of this signaling system is that, since tyrosine phosphorylation of IRS is catalytic, IRS proteins provide a means for signal amplification by eliminating the need for stoichiometric ratios of receptors that directly recruit SH2-containing proteins to their auto-phosphorylation sites.

Do IRS-1 and IRS-2 serve the same cellular functions? The similar tissue distribution may suggest signalling redundancy; however, a number of differences have been documented: mice with targeted disruption of IRS-1 and -2 are beginning to reveal functional differences. In contrast to mice lacking IRS-1 that look normal,[9] IRS-2 knockout mice develop overt diabetes.[10] IRS-1 appears to have its major role in muscle, whereas IRS-2 mediates insulin action in liver, fat, and muscle.[11,12] Knockout mice have shown that disruption of IRS- gene results in compensatory mechanisms affecting the function of the other substrates.[13] Furthermore, while insulin can stimulate IRS-1-associated Grb-2 phosphorylation as well as tyrosine phosphorylation of Shc proteins, nearly no Grb-2 phosphorylation was detected when IRS-2 was analyzed in fetal rat brown adipocytes.[14] Another differential response is related to the finding that IRS-1 displays a more sustained signal than IRS-2.[5]

Correspondence to: Alfonso Colombatti, M.D., Divisione di Oncologia Sperimentale 2, Centro di Riferimento Oncologico, 33081 Aviano, Italy. Tel.: +39-0434-659-365; Fax: +39-0434-659-428; E-mail: acolombatti@cro.it

ISSN 1357–714X print/ISSN 1369–1643 online/02/0200089–08 © Taylor & Francis Ltd
DOI: 10.1080/1357714021000065387

Leiomyosarcomas in soft tissue are relatively rare neoplasms. They show a wide range of behaviours, i.e., nearly benign to severely malignant. Uterine leiomyosarcomas are aggressive tumours with a recurrence rate of 71%,[15] with tumour size being found as a major prognostic parameter only in one study.[16] Few studies have focused on their biology,[17,18] and prognostic factors are currently sought with the aim of recognizing predictors of patient survival. We investigated the possible involvement of IRS-1 and IRS-2 in the determination of the malignant phenotype of three uterine leiomyosarcoma cell lines.

### Materials and methods

### *Antibodies*

Rabbit polyclonal antibodies to Shc and mouse monoclonal antibodies to Grb-2 were purchased from Transduction Laboratories (Lexington, KY, USA). Anti-phosphotyrosine monoclonal antibody (PY20), rabbit polyclonal antibodies to IRS-1, IRS-2 and PI3-K, and goat polyclonal HRP-conjugated antibodies to rabbit or mouse IgG H+L were from Upstate Biotechnology (Lake Placid, NY, USA).

### *Cell lines*

The uterine leiomyosarcoma cell lines SK-LMS-1[19] and SK-UT-1B[20] were purchased from ATCC. DMR cells were isolated from the buffy coat from the peripheral blood of a patient with a relapsed uterine leiomyosarcoma referred to our Institution in May 1997. Within 8–10 days of culture from the 'spongy' clot at the bottom of the flask, adherent transformed cells started growing and were then trasferred twice a week. During the course of these studies DMR cells were used at passage 35 or above; a more complete description of the isolation procedures and other properties of this cell line will be presented in a separate report. SK-LMS-1, SK-UT-1B and DMR were maintained in DMEM supplemented with 10% FCS (Gibco, Paisley, UK), 2 mM glutamine, 100 U/ml penicillin, 500 μg/ml gentamicin, 100 μg/ml streptamicin (Sigma, St. Louis, MO, USA). Cells were cultured at 37°C with 5% $CO_2$.

### *Anchorage-independent growth assay*

To assay the ability of single cells to proliferate while suspended in a semisolid medium, a soft agar assay was used. Cells ($7.5 \times 10^4$ and $3.75 \times 10^4$ cells/well) were seeded in 1 ml complete medium containing 0.3% agarose Type VII Low Gelling Temperature (Sigma) on top of a solid underlay of 2 ml 0.5% agarose in complete medium. Six-well plates were incubated at 37°C with 5% $CO_2$ for up to 10 days and the number of colonies determined macroscopically.

### *Effect of Matrigel on in vitro invasion*

Matrigel, an extract of the murine Engelbreth-Holm-Swarm (EHS) tumour grown in C57/Bl6 mice, was from Collaborative Biochemical Products (Becton-Dickinson Labware, Bedford, MA). Subconfluent cultures were washed with PBS, harvested with trypsin, centrifuged and the pellet ($5 \times 10^3$ cells) was resuspended in 10 μl Matrigel (10 μg/ml, kept at 4°C). The droplets were plated in a six-well plate and the mixtures were incubated at 37°C for 30 min to allow for polymerization. Then DMEM containing 10% FCS was added and the ability of the cells to egress from Matrigel was evaluated taking images at 24 h on a Leica DM IRB inverted microscope (Leica Microsystems Wetzlar GmbH, Wetzlar, Germany).

### *Immunoprecipitation*

SK-LMS-1, SK-UT-1B and DMR cells (after 20 h of serum deprivation) were treated with porcine insulin $10^{-7}$ M (Sigma) for 1, 2, 5, or 10 min at 37°C and subsequently lysed at 4°C in 1 ml of solution containing 50 mM Tris-HCl, pH 7.0, 150 mM NaCl, 5 mM EDTA, 1 mM NaF, 2 mM $NaVO_4$, 1% (w/v) Triton X-100, 100 μg/ml phenylmethylsulfonyl fluoride (PMSF), 0.5% (w/v) sodium deoxycholate, 0.1% (w/v) SDS, 1 μg/ml leupeptin, 1 μg/ml aprotinin, 1 μg/ml pepstatin (RIPA lysis buffer). Cell lysates were clarified by centrifugation at 13,000 rpm for 20 min at 4°C, and the supernatants were transferred to a fresh tube. After protein content determination using the DC Protein Assay (Biorad, Hercules, CA, USA) and BSA as the standard, equal amounts of protein were immunoprecipitated with the corresponding antibodies at 4°C for 16 h, and the immune complexes were collected on Protein A-Sepharose (Amersham-Pharmacia Biotech, Little Chalfont, UK) and resolved on a reduced 10% SDS-PAGE. Cell lysates were precleared with protein A-Sepharose to minimize non-specific binding. As an additional control for non-specific binding, samples were also immunoprecipitated with pre-immune rabbit or mouse IgG.

### *Immunoblotting*

Cell lysates or immunoprecipitated proteins were resolved on SDS-PAGE and transferred to a nitrocellulose filter. Before immunoblotting, non-specific attachment to membranes was blocked using 0.1% Tween-20 and 0.5% BSA in PBS for 1 h; the membranes were then incubated overnight with the indicated antibodies in 20 mM Tris-HCl, pH 7.4, 150 mM NaCl, and 0.1% Tween-20 (TBS). Blots were incubated with HRP-conjugated goat anti-rabbit or goat anti-mouse antibodies (1:1000) and developed using the ECL system (Amersham-Pharmacia Biotech) and only the relevant part of the gel is shown.

*In vivo growth studies*

Eight- to 12-week-old athymic nude mice were purchased from Charles River Italia (Calco, Italy) and housed in laminar flow hoods in sterile cages with filters. Sterile water and rodent chow were provided *ad libitum*. Cells ($6.0 \times 10^5$) in 0.2 ml 0.9% NaCl were inoculated s.c. into the interscapular area. Tumour incidence, latency and growth rate were determined twice a week; tumour diameters were measured with a caliper and the volume ($V$) was calculated according to the formula: $V = 1/2LW^2$ (where $L$ = length, $W$ = width).

### Results

*Invasive capacity in Matrigel and tumorigenesis of leiomyosarcoma cell lines*

To investigate the invasive potential of the SK-LMS-1, SK-UT-1B, and DMR cell lines, a semi-quantitative *in vitro* assay was first applied. In this case $5.0 \times 10^3$ cells were mixed with Matrigel and the gellyfied matrix was incubated at 37°C in DMEM containing 10% FCS. While SK-UT-1B cells migrated extensively from the gel after 20 h of incubation, only a few SK-LMS-1 cells were present outside the gel and DMR did not egress at all (Fig. 1A and data not shown). Next, to have a measure of their real oncogenic capacity *in vivo* the ability to form tumours in nude mice was then investigated. Also in this case a striking difference was noted among the different cell lines: both DMR and SK-UT-1B displayed a consistent ability to form solid tumours already at 10 days, that grew to very large sizes. DMR tumours had a doubling time of about 5 days between 20 and 30 days post-injection, whereas the exponential growth of SK-UT-1B tumours was delayed until day 35 and then the tumours started growing also with a doubling time of 5 days (Fig. 1B). No tumours were detected in mice injected with SK-LMS-1 cells.

*Colonies in agar*

To investigate whether the lack of tumorigenesis of SK-LMS-1 cells might be the consequence of an *in vivo* host effect, the ability to proliferate in an anchorage-independent environment was then assayed *in vitro* in semi-solid agar. Also in this case both DMR and SK-UT-1B cells formed a similar number of colonies (Fig. 1C) with a mean cell number >30 cells per colony. Although it is generally accepted that DNA synthesis and cellular proliferation can be stimulated very effectively by IGFs, insulin and its receptor(s) can also stimulate growth and metabolism in tumour cells. To determine whether the growth in agar of SK-LMS-1 could be stimulated by supplementation with insulin, colony formation was investigated in the presence of a physiological dose ($10^{-7}$ M) of insulin. No substantial differences compared to cells grown in the absence of the stimulus were detected when DMR or SK-UT-1B cells were investigated. Similarly, supplementation with insulin did not increase colony formation, neither the numbers nor the sizes of the colonies, also when SK-LMS-1 were stimulated with insulin (data not shown).

*Differential expression and activation of IRS-1 and IRS-2*

To rule out the possibility that SK-LMS-1 cells had totally lost sensitivity to insulin, the basal expression of IRS-1 and IRS-2, the major substrates of the insulin receptor, was first investigated by immunoblotting with specific antibodies in cell lysates. SK-LMS-1 cells expressed high levels of IRS-1, whereas in both DMR and SK-UT-1B cells IRS-1 was barely visible (Fig. 2). Conversely, very little IRS-2 was present in SK-LMS-1 while DMR and SK-UT-1B were highly positive for IRS-2. Next, the insulin-dependent activation was measured at different time intervals after insulin stimulation by immunoblotting with anti-phosphotyrosine antibodies on cell lysates (Fig. 3A). The lowest effective dose of insulin for the present cell lines has been preliminarly determined based on cell duplication rate to be around $10^{-7}$ M. A transient increase in phosphorylation was evident between 1 and 5 min post-activation for bands in the range of 160–180 kDa, compatible with the migration of IRS-1 and IRS-2. To confirm that the bands identified corresponded to authentic IRS-1 and IRS-2, cell lysates from each cell line were first immunoprecipitated with either anti-IRS-1 or IRS-2 antibodies and then the blotted immunoprecipitates were probed with anti-phosphotyrosine antibodies (Fig. 3B). Consistent with the results of immunoblotting on total cell extracts (Fig. 2), only IRS-1 was immunoprecipitated from SK-LMS-1 cells that was not phosphorylated in the absence of insulin treatment. After 1 min treatment, a rapid increase of IRS-1 phosphorylation was detected; conversely, no activity was detected when the SK-LMS-1 cell lysate was immunoprecipitated with anti IRS-2 antibodies and probed with anti-phosphotyrosine antibodies (data not shown). Insulin-dependent IRS-2 activation was detected instead in both DMR and SK-UT-1B cell lysates at 1 min. At 2 min of insulin stimulation, phosphorylation had declined significantly in SK-UT-1B, whereas it remained constant in DMR and even increased in SK-LMS-1.

*Downstream signaling*

From the preceding series of experiments, it was clear that the most oncogenic cell lines expressed exclusively IRS-2, whereas the non-tumourigenic SK-LMS-1 cells expressed only IRS-1. Furthermore, upon insulin stimulation the respective principal

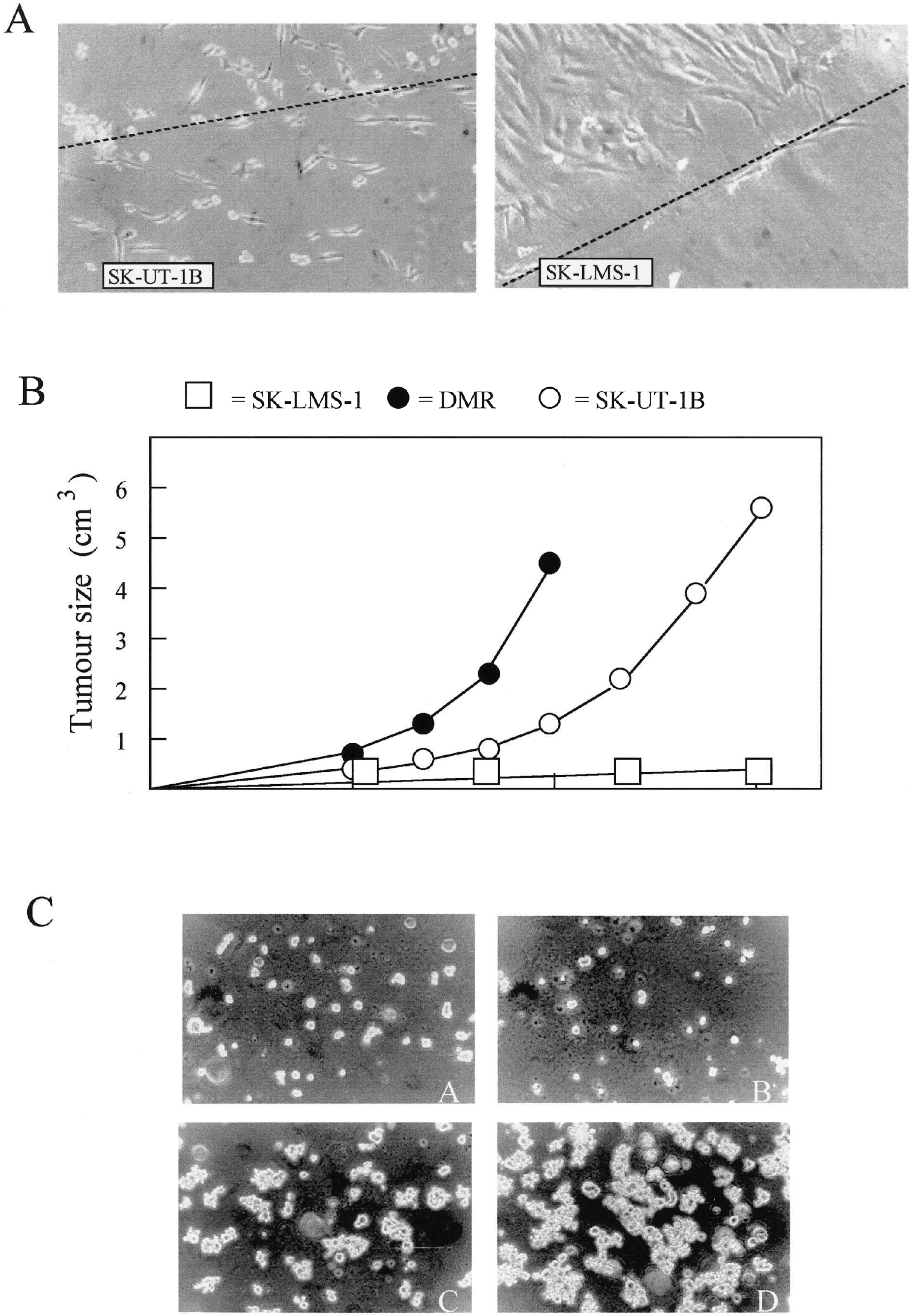

**Fig. 1.** *Invasive and growth capacity in vitro and tumour development in vivo. (A) Matrigel invasion. Cells were included in cold Matrigel, plated, incubated for 20 h at 37°C and then photographed under phase contrast. The dotted line indicates the hedge of the Matrigel drop. (B) Tumour development in nude mice. Mice were injected in the interscapular area with $6.0 \times 10^5$ cells of the indicated cell lines and tumour mass measured at different time intervals. (C) Anchorage-independent growth. A total of $3.75 \times 10^4$ cells/well was seeded in semisolid agar plates in complete medium and colony formation was evaluated under phase contrast at 5 (A,C,D) and 10 (B) days. (A,B) SK-LMS-1; (C) SK-UT-1B; and (C) DMR.*

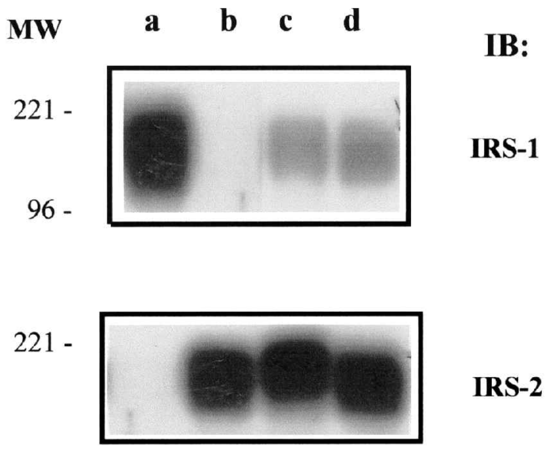

**Fig. 2.** *Basal expression of IRS-1 and IRS-2. SK-LMS-1 (a), SK-UT-1B (b), DMR (c), and mammary carcinoma MDA (d) cells were lysed in RIPA lysis buffer, clarified by centrifugation and equal amounts of proteins (75 µg) were resolved by SDS-PAGE and immunoblotted with antisera specific for IRS-1 and IRS-2. The filter was incubated first with IRS-1 antibody, stripped and then incubated with the IRS-2 antibody. Exposure times were the same. Only the relevant part of the blot is shown. IB, immunoblotting; MW, molecular weight (×$10^{-3}$).*

substrate of the insulin receptor tyrosine kinase expressed within a given cell was activated. This raised the question as to whether the downstream signaling cascade would be different depending upon which IRS was expressed and activated. Thus, the role of PI3-kinase, a central molecule in the insulin signaling was first analysed. Cell lysates of insulin-stimulated SK-LMS-1, and SK-UT-1B or DMR were immunoprecipitated with anti-IRS-1 or -IRS-2 antibodies, respectively, and the blotted precipitates were probed with anti-PI3-kinase antibody. Already at 1 min a strong association was detected in all cell samples that increased slightly at 2 min (Fig. 4A). Thus, no difference between IRS-1 and IRS-2 expressing cells was apparent in PI3-kinase association.

Next, the Ras/MAPK pathway that is known to be involved in determining cell proliferation and locomotion in several tumour systems[21] was analysed. In fact, this pathway might have been differently activated in the less malignant SK-LMS-1 versus the more malignant SK-UT-1B and DMR cells. However, after repeated attempts to demonstrate a direct association between IRS-1 or IRS-2 and Grb2 as a way to activate the Ras pathway, no association was detected notwithstanding a strong expression of Grb2 in all cell lines (data not shown). Another way in which the Ras/MAPK pathway could be differently activated between SK-LMS-1 and SK-UT-1B or DMR cells is via association of Shc with Grb2. An increase in the phosphorylation of the 52-kDa species of Shc was evident at 2 min and remained constant at 5 min in SK-LMS-1 and SK-UT-1B cells, whereas in DMR the phosphorylation peaked at 5 min and then returned at normal levels at 10 min (Fig. 4B). The association with Grb2 was demonstrable for all three cell lines starting at 2 min; then, it remained constant up to 5 min in SK-LMS-1, whereas it peaked at 5 min in SK-UT-1B and DMR (Fig. 4B).

### Discussion

IRS-1 and IRS-2 represent the most upstream molecules in the signal transduction cascade mediated by insulin and other ligands. Several intermediate signaling events including the activation of PI3-kinase pathway,[22] have been implicated in insulin- and IGF-1-stimulated mitogenesis. However, IRSs are essential not only for insulin-induced mitogenic effects but may even contribute to epithelial carcinogenesis.[23–25] Therefore, notwithstanding the legitimate concern that the connection between the differential expression of IRS-1 and IRS-2 events and events that materialize days (colonies in agar) or even weeks (tumours) later, we tested the hypothesis that constitutive high expression of these molecules may result in a more malignant and tumourigenic phenotype also in leiomyosarcomas; in addition, it was analysed whether IRS genes activation triggers different downstream cascades that might be associated with a differential malignant behaviour.

At least two lines of evidence led us to suggest that IRS-2 could contribute to enhanced malignancy in uterine leiomyosarcoma cell lines. First, IRS-2 was expressed in SK-UT-1B and DMR cell lines that displayed strong anchorage-independent growth in agar and were tumourigenic *in vivo*, whereas IRS-1 was expressed in the non-tumourigenic SK-LMS-1 cell line. In addition, SK-LMS-1 did not form colonies in agar, nor were these cells able to egress out of Matrigel, whereas both SK-UT-1B and DMR cells formed large colonies in agar, and SK-UT-1B migrated extensively out of Matrigel indicating that malignant characteristics other than *in vivo* tumorigenesis were associated with IRS-2-expressing cells. The finding that DMR cells did not migrate out of Matrigel might depend on the lack of expression of the appropriate integrins (P. Spessotto *et al.*, unpublished data). Second, in both SK-UT-1B and DMR cell lines insulin enhanced tyrosine phosphorylation of IRS-2 and IRS-2-associated PI3-kinase. Since preliminary insulin dose-response relationship experiments had indicated that the present cell lines responded by an increased proliferation at doses around or higher than $10^{-7}$ M, we have not further investigated whether lower concentrations might still trigger any signaling molecule.

Thus, insulin at $10^{-7}$ M similarly stimulated tyrosine phosphorylation of IRS-1 and IRS-1-associated PI3-kinase in SK-LMS-1 cells, indicating that the major key downstream signaling switch[22] was not differentially activated in IRS-1- and IRS-2-expressing smooth muscle cell tumours. Furthermore, also the Ras/MAPK pathway did not seem one of the major direct targets of insulin stimulation

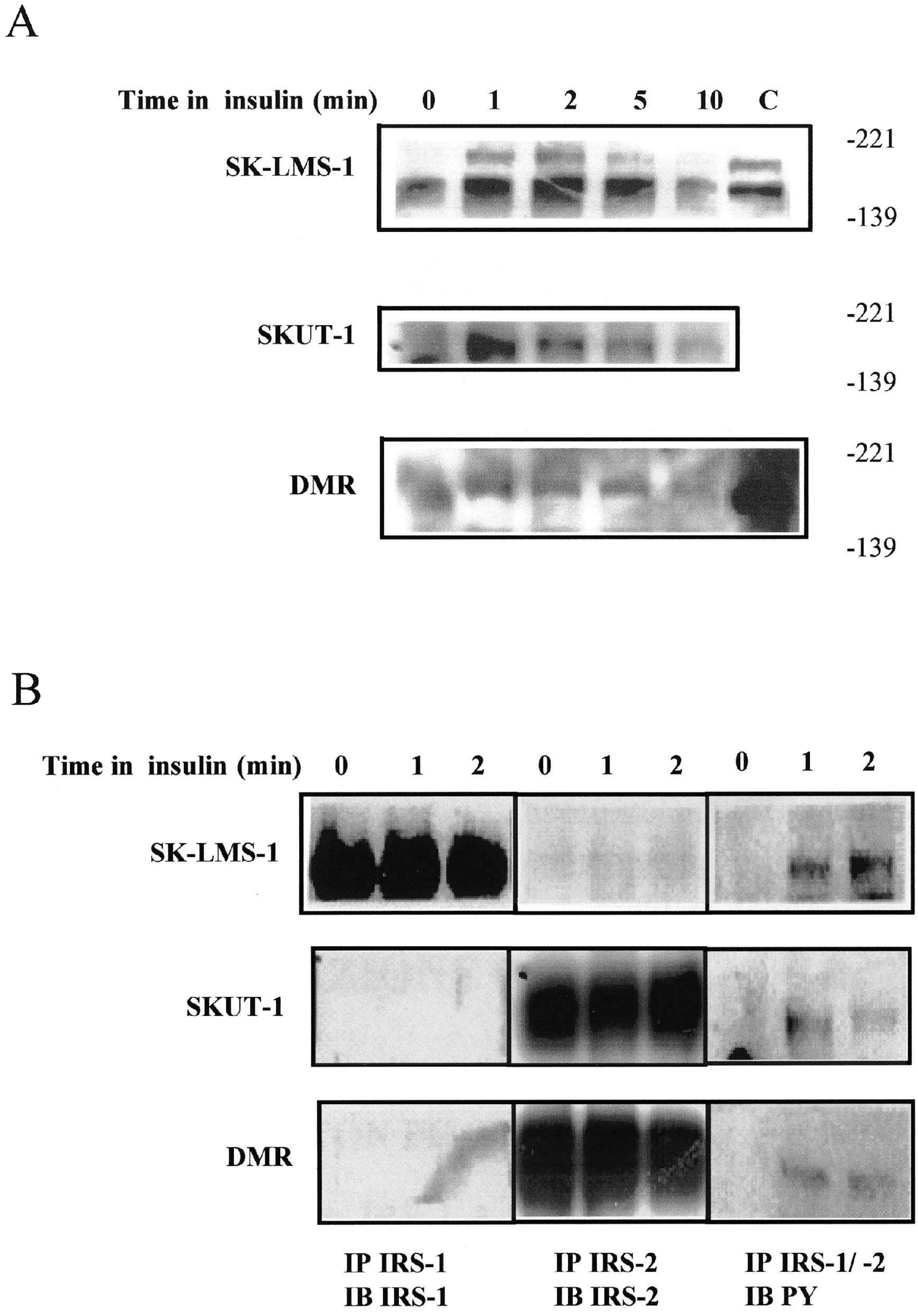

**Fig. 3.** *Effects of insulin treatment on the phosphorylation of IRS-1 and IRS-2. Exponentially growing cells were seeded in serum-free medium for 24 h before stimulation with insulin (0.1 μM) for various lengths of time (0–10 min). (A) Immunoblotting of cell lysates. Cells were lysed in RIPA lysis buffer, clarified by centrifugation and equal amounts of proteins (75 μg) were resolved by 10% SDS-PAGE and immunoblotted with antibody PY20 specific for phosphotyrosine. The PY20 antibody labels bands in the range of 160–180 kDa corresponding to IRS-1 and IRS-2. (B) Immunoprecipitation and immunoblotting. Clarified cell lysates (1 mg of protein) were immunoprecipitated overnigth with either IRS-1 or IRS-2 antibodies and then the washed pellets were resolved by 10% SDS-PAGE and immunoblotted with IRS-1, IRS-2, and PY20 antibodies. Only the relevant part of the blot is shown. (C) Epidermoid carcinoma A431 cells stimulated with 0.1 μM insuline for 1.5 min; IB, immunoblotting; IP, immunoprecipitation; MW, molecular weight (×10$^{-3}$).*

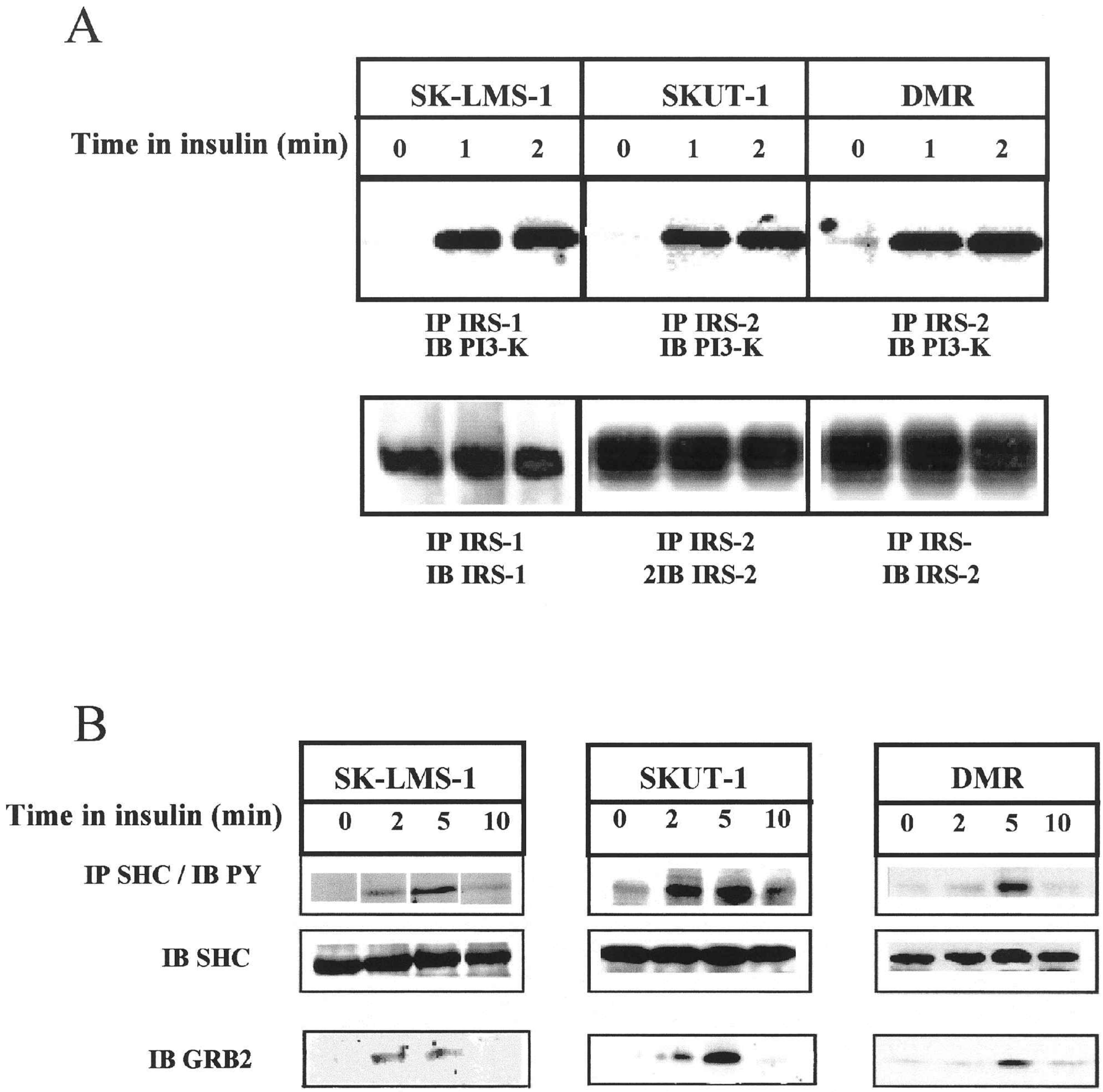

**Fig. 4.** *Insulin-dependent signaling in uterine leiomyosarcoma cell lines. (A) Association of PI 3-kinase with IRS-1 and IRS-2. (B) Activation of Shc and association between Shc and Grab2. Exponentially growing cells were seeded in serum-free medium for 24 h before stimulation with insulin (0.1 μM) for various lengths of time (0–2 min or 0–10 min). Clarified lysates (1 mg of protein) were immunoprecipitated overnigth with either IRS-1 or IRS-2 or with Shc antibodies and then the washed pellets were resolved by 10% SDS-PAGE and immunoblotted with PI 3-kinase antibody (A) or with PY20 anti-phosphotyrosine, stripped and reprobed with Grb2 antibodies (B). Only the relevant part of the blot is shown. IB, immunoblotting, IP, immunoprecipitation.*

in either IRS-1- or IRS-2-positive leiomyosarcomas. In this case, the explanation for the lack of correlation could be that the role played by IRSs in MAP kinase activation differs from tissue to tissue[26] and this type of tumours belong to a tissue histotype that is not particularly active in this respect.

Finally, the downstream signaling following insulin-dependent IRS activation demonstrated that Shc was activated and associated with Grb2 in both IRS-1 and IRS-2 expressing cell lines. Thus, the finding that the major transformation-related signaling pathways activated by IRS-1 and IRS-2 resulted in being highly similar if not identical makes it unlikely that the different biological behaviours of the uterine leiomyosarcomas examined in the present study are directly linked to the insulin pathway, as shown in other types of tumours.[24,27–29] The minor differences in the duration and/or in the timing of peak activities (phosphorylation and/or association between Shc and Grb2) detected do not seem to be relevant in view of the fact that they were not specifically related to either IRS-1 or IRS-2 expression. Whether differences between IRS-1- and/or IRS-2-expressing cells in PI3-kinase-dependent pathways not examined here might be responsible for the distinct phenotypes of leiomyosarcomas is a matter of further investigation.

### Acknowledgments

This work was supported by grants (to A.C.) from MURST-Cofin 1999, FSN-1999, and Fondo Dipartimentale. We are indebted to Maria Teresa Mucignat for her expert technical assistance. The present address of Dr. Pietro Russo is Medical Division and Regulatory Affairs, S.I.F.I. S.p.A., Italy.

### References

1. Kahn CR. Insulin action, diabetogenes, and the cause of type II diabetes. *Diabetes* 1994; 43: 1066–84.
2. Lavan BE, Fantin VR, Chang ET, Lane WS, Keller SR, Lienhard GE. A novel 160-kDa phosphotyrosine protein in insulin-treated embryonic kidney cells is a new member of the insulin receptor substrate family. *J Biol Chem* 1997; 272: 21403–7.
3. Sun XJ, Rothenberg P, Kahn CR, Backer JM, Araki E, Wilden PA, Cahill DA, Goldstein BJ, White MF. Structure of the insulin receptor substrate IRS-1 defines a unique signal transduction protein. *Nature* 1991; 352: 73–7.
4. Sun XJ, Wang LM, Zhang Y, Yenush L, Myers MG, Glasheen E, Lane WS, Pierce JH, White MF. Role of IRS-2 in insulin and cytokine signalling. *Nature* 1995; 377: 173–77.
5. Inoue G, Cheatham B, Emkey R, Kahn CR. Dynamics of insulin signaling in 3T3-L1 adipocytes. Differential compartmentalization and trafficking of insulin receptor substrate (IRS)-1 and IRS-2. *J Biol Chem* 1998; 273: 11548–55.
6. Clark SF, Martin S, Carozzi AJ, Hill MM, James DE. Intracellular localization of phosphatidylinositide 3-kinase and insulin receptor substrate-1 in adipocytes: potential involvement of a membrane skeleton. *J Cell Biol* 1998; 140: 1211–25.
7. Clark SF, Molero JC, James DE. Release of insulin receptor substrate proteins from an intracellular complex coincides with the development of insulin resistance. *J Biol Chem* 2000; 275: 3819–26.
8. Whitehead JP, Clark SF, Urso B, James DE. Signalling through the insulin receptor. *Curr Opin Cell Biol* 2000; 12: 222–28.
9. Araki E, Lipes MA, Patti ME, Bruning JC, Haag B, Johnson RS, Kahn CR. Alternative pathway of insulin signalling in mice with targeted disruption of the IRS-1 gene. *Nature* 1994; 372: 186–90.
10. Withers DJ, Gutierrez JS, Towery H, Burks DJ, Ren JM, Previs S, Zhang Y, Bernal D, Pons S, Shulman GI, Bonner-Weir S, White MF. Disruption of IRS-2 causes type 2 diabetes in mice. *Nature* 1998; 391: 900–4.
11. Previs SF, Withers DJ, Ren JM, White MF, Shulman GI. Contrasting effects of IRS-1 versus IRS-2 gene disruption on carbohydrate and lipid metabolism in vivo. *J Biol Chem* 2000; 275: 38990–4.
12. Kido Y, Burks DJ, Withers D, Bruning JC, Kahn CR, White MF, Accili D. Tissue-specific insulin resistance in mice with mutations in the insulin receptor, IRS-1, and IRS-2. *J Clin Invest* 2000; 105: 199–205.
13. Yamauchi T, Tobe K, Tamemoto H, Ueki K, Kaburagi Y, Yamamoto-Honda R, Takahashi Y, Yoshizawa F, Aizawa S, Akanuma Y, Sonenberg N, Yazaki Y, Kadowaki T. Insulin signalling and insulin actions in the muscles and livers of insulin-resistant, insulin receptor substrate 1-deficient mice. *Mol. Cell Biol* 1996; 16: 3074–84.
14. Valverde AM, Lorenzo M, Pons S, White MF, Benito M. Insulin receptor substrate (IRS) proteins IRS-1 and IRS-2 differential signaling in the insulin/insulin-like growth factor-I pathways in fetal brown adipocytes. *Mol Endocrinol* 1998; 12: 688–97.
15. Major FJ, Blessing JA, Silverberg SG, Morrow CP, Creasman WT, Currie JL, Yordan E, Brady MF. Prognostic factors in early-stage uterine sarcoma. A Gynecologic Oncology Group study. *Cancer* 1993; 71: 1702–9.
16. Evans HL, Chawla SP, Simpson C, Finn KP. Smooth muscle neoplasms of the uterus other than ordinary leiomyoma. A study of 46 cases, with emphasis on diagnostic criteria and prognostic factors. *Cancer* 1988; 62: 2239–47.
17. Robboy SJ, Bentley RC, Butnor K, Anderson MC. Pathology and pathophysiology of uterine smooth-muscle tumours. *Environ Health Perspect* 2000; 108: 779–84.
18. Clement PB. The pathology of uterine smooth muscle tumours and mixed endometrial stromal-smooth muscle tumours: a selective review with emphasis on recent advances. *Int J Gynecol Pathol* 2000; 19: 39–55.
19/ Madsen WE, Gupta TK, Walker MJ. The influence of glucocorticoids on the growth of a human leiomyosarcoma cell line SK-LMS-1. *Int J Cancer* 1989; 44: 1034–40.
20. Chen TR. SK-UT-1B, a human tumourigenic diploid cell line. *Cancer Genet Cytogenet* 1988; 33: 77–81.
21. Lewis TS, Shapiro PS, Ahn NG. Signal transduction through MAP kinase cascades. *Adv Cancer Res* 1998; 74: 49–139.
22. Shepherd PR, Withers DJ, Siddle K. Phosphoinositide 3-kinase: the key switch mechanism in insulin signalling. *Biochem J* 1998; 333: 471–90.
23. Surmacz E, Burgaud JL. Overexpression of insulin receptor substrate 1 (IRS-1) in the human breast cancer cell line MCF-7 induces loss of estrogen requirements for growth and transformation. *Clin Cancer Res* 1995; 1: 1429–36.
24. Kornmann M, Maruyama H, Bergmann U, Tangvoranuntakul P, Beger HG, White MF, Korc M. Enhanced expression of the insulin receptor substrate-2 docking protein in human pancreatic cancer. *Cancer Res* 1998; 58: 4250–4.
25. Tanaka S, Ito T, Wands JR. Neoplastic transformation induced by insulin receptor substrate-1 overexpression requires an interaction with both Grb2 and Syp signaling molecules. *J Biol Chem* 1996; 271: 14610–6.
26. Chen D, Van Horn DJ, White MF, Backer JM. Insulin receptor substrate 1 rescues insulin action in CHO cells expressing mutant insulin receptors that lack a juxtamembrane NPXY motif. *Mol Cell Biol* 1995; 15: 4711–7.
27. Nerbass D, Klimek F, Bannasch P. Overexpression of insulin receptor substrate-1 emerges early in hepatocarcinogenesis and elicits preneoplastic hepatic glycogenesis. *Am J Pathol* 1998; 152: 341–5.
28. Schnarr B, Strunz K, Ohsam J, Benner A, Wacker J, Mayer D. Down-regulation of insulin-like growth factor-I receptor and insulin receptor substrate-1 expression in advanced human breast cancer. *Int J Cancer* 2000; 89: 506–13.
29. Weng LP, Smith WM, Brown JL, Eng C. PTEN inhibits insulin-stimulated MEK/MAPK activation and cell growth by blocking IRS-1 phosphorylation and IRS-1/Grb-2/Sos complex formation in a breast cancer model. *Hum Mol Genet* 2001; 10: 605–16.
